# Construction and immunogenicity of a trypsin-independent porcine epidemic diarrhea virus variant

**DOI:** 10.3389/fimmu.2023.1165606

**Published:** 2023-03-24

**Authors:** Mingxiang Li, Yiye Zhang, Yuxin Fang, Shaobo Xiao, Puxian Fang, Liurong Fang

**Affiliations:** ^1^ State Key Laboratory of Agricultural Microbiology, College of Veterinary Medicine, Huazhong Agricultural University, Wuhan, China; ^2^ Key Laboratory of Preventive Veterinary Medicine in Hubei Province, The Cooperative Innovation Center for Sustainable Pig Production, Wuhan, China

**Keywords:** porcine epidemic diarrhea virus (PEDV), trypsin-independent, chimeric virus, immunogenicity, neutralizing antibodies

## Abstract

Porcine epidemic diarrhea virus (PEDV) is a re-emerging enteropathogenic coronavirus that causes high mortality in neonatal piglets. The addition of trypsin plays a crucial role in the propagation of PEDV, but also increases the complexity of vaccine production and increases its cost. Previous studies have suggested that the S2′ site and Y976/977 of the PEDV spike (S) protein might be the determinants of PEDV trypsin independence. In this study, to achieve a recombinant trypsin-independent PEDV strain, we used trypsin-dependent genotype 2 (G2) PEDV variant AJ1102 to generate three recombinant PEDVs with mutations in S (S2′ site R894G and/or Y976H). The three recombinant PEDVs were still trypsin dependent, suggesting that the S2′ site R894 and Y976 of AJ1102 S are not key sites for PEDV trypsin dependence. Therefore, we used AJ1102 and the classical trypsin-independent genotype 1 (G1) PEDV strain JS2008 to generate a recombinant PEDV carrying a chimeric S protein, and successfully obtained trypsin-independent PEDV strain rAJ1102-S2′_JS2008_, in which the S2 (amino acids 894–1386) domain was replaced with the corresponding JS2008 sequence. Importantly, immunization with rAJ1102-S2′_JS2008_ induced neutralizing antibodies against both AJ1102 and JS2008. Collectively, these results suggest that rAJ1102-S2′_JS2008_ is a novel vaccine candidate with significant advantages, including no trypsin requirement for viral propagation to high titers and the potential provision of protection for pigs against G1 and G2 PEDV infections.

## 1 Introduction

Porcine epidemic diarrhea virus (PEDV) causes highly contagious and acute viral enteritis in newborn piglets (1, 2). It is an enveloped, positive-sense, single-stranded RNA virus belonging to the genus *Alphacoronavirus* within the family *Coronaviridae*. Its genome is approximately 28 kb in length, encoding 16 mature nonstructural proteins and four structural proteins (spike glycoprotein [S], small envelope protein [E], membrane glycoprotein [M], and nucleocapsid protein [N]) (3). Classical PEDV (genotype 1, G1), CV777, was first isolated in Belgium in 1978 (4). In 2010, an emerging PEDV variant (genotype 2, G2) resulted in a large-scale outbreak of porcine epidemic diarrhea (PED) in China, with almost 100% mortality in suckling piglets, leading to tremendous economic losses and sparking tremendous attention (5–7). PEDV variants were subsequently reported in many countries in Asia, including South Korea (8), Japan (9), Vietnam (10), Thailand (11), and the Philippines (12), and in the main pig-raising countries and regions in the Americas (13–16) and Europe (17–20). At present, this PEDV variant is a leading pathogenic cause of piglet diarrhea, and poses an enormous threat to the global pig industry.

No specific drugs for the treatment of PED are currently available commercially, so biosafety measures and immunization are still the main strategies for the prevention and control of PEDV (21). PEDV is a coronavirus that requires the presence of an exogenous protease, trypsin, in the cell culture medium for its propagation (22). Cell cultures require the presence of serum, but serum has a negative effect on the activity of trypsin. Therefore, the cells must be washed with phosphate-buffered saline (PBS) and then added to a medium containing trypsin before they are inoculated with PEDV. However, using a trypsin-dependent PEDV as a vaccine strain complicates the process and increases the cost of vaccine production. Interestingly, there have been reports of the transformation of trypsin-dependent strains into trypsin-independent strains by their serial passage or the addition of glycochenodeoxycholic acid to the cell culture *in vitro*, including PEDV strains DR13, CV777, 85-7, 8aa and KPEDV-9 (23–26). However, the detailed mechanisms of the trypsin-independent or -dependent activities of PEDV are largely unknown. Understanding the mechanisms of trypsin-dependent events and generating trypsin-independent strains should facilitate the development of a PEDV vaccine.

The PEDV spike (S) protein is an important surface protein that plays a crucial role in viral infection. Multiple studies have shown that the addition of trypsin to the cell culture is related to the cleavage of the PEDV S protein, indicating that S protein is the trypsin-dependence determinant (24, 27, 28). In the S proteins of many coronaviruses, including severe acute respiratory syndrome coronavirus (SARS-CoV), Middle East respiratory syndrome coronavirus (MERS-CoV), mouse hepatitis virus (MHV), and infectious bronchitis virus (IBV), an alternative cleavage site (S2′) located upstream from the putative fusion peptide has been extensively described (29–32). A previous study of PEDV also mapped the genetic determinant of its trypsin dependence to the S2′ site (R890) just upstream from the putative fusion peptide (28). However, another study demonstrated that the PEDV S2′ site (R895) is crucial for viral replication and infection but is not a prerequisite for trypsin dependence (26). A similar result was also reported by Tan et al. (27). Sequence alignments from several studies have also shown that another site, Y976/977, in the S protein close to the heptad repeat region 1 (HR1) may be the determinant of the PEDV trypsin-independent phenotype and may be a crucial site for PEDV adaptation to Vero cells (24, 26, 33). However, this remains to be verified with further experiments.

In the present study, we used an infectious clone and the reverse genetics of PEDV strain AJ1102, a trypsin-dependent G2 PEDV variant, to demonstrate that S2′ site R894 and Y976 of the AJ1102 S protein are not trypsin-dependence determinants. We then used PEDV strain JS2008, a classical trypsin-independent G1 PEDV strain, and strain AJ1102 to generate chimeric AJ1102 by replacing the S2 subdomain (amino acids, aa 894-1386) with the corresponding JS2008 sequence, designating it rAJ1102-S2′_JS2008_. We showed that rAJ1102-S2′_JS2008_ can be stably passaged independently of trypsin and has a higher viral titer compared to AJ1102 *in vitro*. Importantly, rAJ1102-S2′_JS2008_ induces neutralizing antibodies against both PEDV strains AJ1102 and JS2008.

## 2 Materials and methods

### 2.1 Ethics statement

All procedures involving animal experiments were reviewed, approved, and conducted in strict accordance with the Animal Experimental Ethical Inspection of Laboratory Animal Centre, Huazhong Agricultural University (Ethics Approval Number: HZAUSW-2023-0001).

### 2.2 Cells, viruses, and antibodies

Vero cells (ATCC CCL-81) were maintained in Dulbecco’s modified Eagle’s medium (DMEM; Gibco, Grand Island, NY), containing 10% fetal bovine serum (FBS) at 37°C in a 5% CO_2_ humidified atmosphere. PEDV strain AJ1102, a highly virulent PEDV variant (G2) isolated from a neonatal piglet with acute diarrhea in China in 2011 (5), was propagated in Vero cells supplemented with trypsin (7.5 μg/mL). PEDV strain JS2008, a classical trypsin-independent virulent PEDV isolate (G1), kindly provided by Dr. Bin Li at Jiangsu Academy of Agricultural Sciences (Nanjing, Jiangsu Province, China), was propagated in Vero cells in medium without trypsin (34). The monoclonal antibody (mAb) directed against PEDV N protein is stored in our laboratory, as described previously (35).

### 2.3 Generation of recombinant viruses

Recombinant PEDVs with a single point mutation (S2′ site R894G or Y976H) or double mutation (R894G/Y976H) in the AJ1102 S protein and the chimeric S gene sequence from classical trypsin-independent PEDV isolate JS2008 were constructed with the CRISPR/Cas9 technology, as described previously (36). Briefly, two specific primers (sgRNA-S2′a and sgRNA-S2′b) targeting the upstream and downstream sequences of the fragment of interest, respectively, were designed and synthesized. Overlapping PCR products amplified respectively with primers of sgRNA-S2′a/S2′b and reverse primer scaffold oligo were used as temples to generate sgRNA-a and sgRNA-b (**Table 1**). pBAC-AJ1102 was then cleaved into a linearized BAC vector by the *in vitro* addition of Cas9, sgRNA-a, and sgRNA-b. At the same time, fragments containing one of the S gene sequences with a specific mutation were generated with overlapping PCR using the indicated primer pairs (PEDV-S2′F/Mid-S_R894G_-R and Mid-S_R894G_-F/PEDV-S2′R, PEDV-S2′F/Mid-S_Y976H_-R and Mid-S_Y976H_-F/PEDV-S2′R) (**Table 1**) and individually ligated into the purified linearized BAC vector by homologous recombination, generating the recombinant BAC plasmids. Similarly, fragments containing chimeric S genes were generated with primer pairs (PEDV-S2′F/Mid-S_993_-R and Mid-S_993_-F/PEDV-S2′R, PEDV-S2′F/Mid-S_R894G_-R and Mid-S_R894G_-F/PEDV-S2′R) (**Table 1**). Vero cells seeded in 12-well plates (2 µg/well) and grown to 80% confluence were transfected with one or other of these recombinant plasmids using Lipofectamine^®^ 3000 (Invitrogen). At 6 h posttransfection, the medium was replaced with DMEM containing 7.5 μg/mL trypsin or 2% FBS. The transfected cells were cultured at 37°C in a humidified atmosphere containing 5% CO_2_ and observed daily with a fluorescence microscope (Nikon) for the appearance of a cytopathic effect (CPE).

**Table 1:** Primers used for the construction of recombinant PEDVs.

Primer	Sequence (5′- 3′)
sgRNA-S2′a	TTCTAATACGACTCACTATAGCATCTGACACTACTATCAATGTTTTAGAGCTAGA
sgRNA-S2′b	TTCTAATACGACTCACTATAGGCCACGTGCAGTGATGTTTCTGTTTTAGAGCTAGA
scaffold oligo	AAAAGCACCGACTCGGTGCCACTTTTTCAAGTTGATAACGGACTAGCCTTATTTTAACTTGCTATTTCTAGCTCTAAAAC
PEDV-S2′F	CATCTGACACTACTATCAAT
PEDV-S2′R	CGTGTATTGAAAAAGTCCAAG
Mid-S_R894G_-F	AGTGGCAGGGTGGTACAAAAAGGGTCTTTTATTGAAGACCTGC
Mid-S_R894G_-R	GCAGGTCTTCAATAAAAGACCCTTTTTGTACCACCCTGCCACT
Mid-S_Y976H_-F	GCGGCATTGCCTTTTAGCGATGCTGTTCAAGCGAGACTGAATTATC
Mid-S_Y976H_-R	CAGTCTCGCTTGAACAGCATCGCTAAAAGGCAATGCCGCTG
Mid-S_993_-F	TACAGACGGATGTTCTACAGCGCAACCAGCAATTGCTTGC
Mid-S_993_-R	GCAAGCAATTGCTGGTTGCGCTGTAGAACATCCGTCTGTA

### 2.4 Indirect immunofluorescence assay

Vero cells seeded in 24-well plates were infected with PEDV for 24 h. The cells were then fixed with 4% paraformaldehyde for 15 min and permeabilized with cold methanol for 15 min at room temperature. They were then washed three times with PBS and blocked with 5% bovine serum albumin for 1 h. After three washes with PBS, the cells were incubated with an anti-PEDV N mAb for 1 h, and then with fluorescein isothiocyanate (FITC)-conjugated goat anti-mouse IgG antibody and 0.01% 4′,6-diamidino-2-phenylindole (DAPI). After the samples were washed three times with PBS, fluorescent images were visualized with a fluorescence microscope (Nikon).

### 2.5 Plaque assay

Monolayers of Vero cells in six-well plates were incubated with 500 μL of 10-fold serially diluted viral samples for 1 h at 37°C with periodic gentle rocking. The treated cells were washed three times with DMEM to remove unabsorbed virus, and then overlain with 1 mL of DMEM containing 1.5% methylcellulose and 7.5 μg/mL trypsin. After incubation at 37°C for 48 h, the cells were fixed with 4% paraformaldehyde and stained with 0.1% crystal violet.

### 2.6 Viral multistep growth curve construction

Vero cells in 12-well plates were inoculated with PEDV at a multiplicity of infection (MOI) of 0.1 in the presence or absence of trypsin. The cell supernatants were collected at different time points (6, 12, 18, 24, 30, 36, 42, and 48 h post-infection (hpi)) and subjected to a median tissue culture infective dose (TCID_50_) assay (37).

### 2.7 Neutralization test

The serum-neutralizing antibody titers were determined with a virus neutralization test in 96-well cell culture plates. Briefly, Vero cells were grown to 2 × 10^4^/well in 96-well tissue culture plates for 1 day. The viral stock was diluted with serum-free DMEM to 200 TCID_50_ in a 50-μL volume. The diluted virus was then mixed with 50 μL of two-fold serial dilutions of each inactivated serum sample in a 96-well plate and incubated at 37°C for 1 h. The Vero cells were inoculated with the mixture and incubated at 37°C for 1 h. After the mixture was removed, the cells were thoroughly rinsed three times with PBS and maintained in virus growth medium at 37°C in a 5% CO_2_ incubator. After incubation for 48 h, the serum neutralization (SN) titer was determined as the reciprocal value of the highest serum dilution that inhibited the PEDV-specific CPE.

### 2.8 Immunization of piglets with inactivated recombinant PEDVs

Twenty-five 28-day-old piglets, born from transmissible gastroenteritis virus (TGEV)- and PEDV-negative sows, were randomly divided into five groups (**Table 2**). Four groups of piglets (n = 5 per group) were immunized intramuscularly in the neck with 2 mL of inactivated wild-type or recombinant PEDV containing 50% adjuvant (Montanide™ ISA 201 ^®^, SEPPIC, France), followed by a booster immunization 2 weeks later. Final bleeding was performed 8 weeks after the last immunization. The control group of piglets (n = 5) were immunized with DMEM and 50% adjuvant. Each serum sample was tested for SN titers against AJ1102 and JS2008, as described above.

**Table 2:** Inoculation dosage of individual group of pig with different PEDVs.

Group	Vaccination	Immunization dose
1	AJ1102	10^6.5^TCID_50_/mL
2	rAJ1102	10^6.5^TCID_50_/mL
3	JS2008	10^6.5^TCID_50_/mL
4	rAJ1102-S2′_JS2008_	10^6.5^TCID_50_/mL
5	DMEM	–

### 2.9 Statistical analysis

Statistical analysis of the data was performed with the GraphPad Prism 8.4.3 software (San Diego, CA, USA), using Student’s *t* test or two-way analysis of variance (ANOVA) for multiple comparisons. Error bars indicate standard deviations. The level of significance is expressed as *P < 0.05, **P < 0.01, ***P < 0.001, or ns: not significant.

## 3 Results

### 3.1 Sites R894 and Y976 of S protein are not determinants of trypsin dependence of strain AJ1102

**Figure 1:**
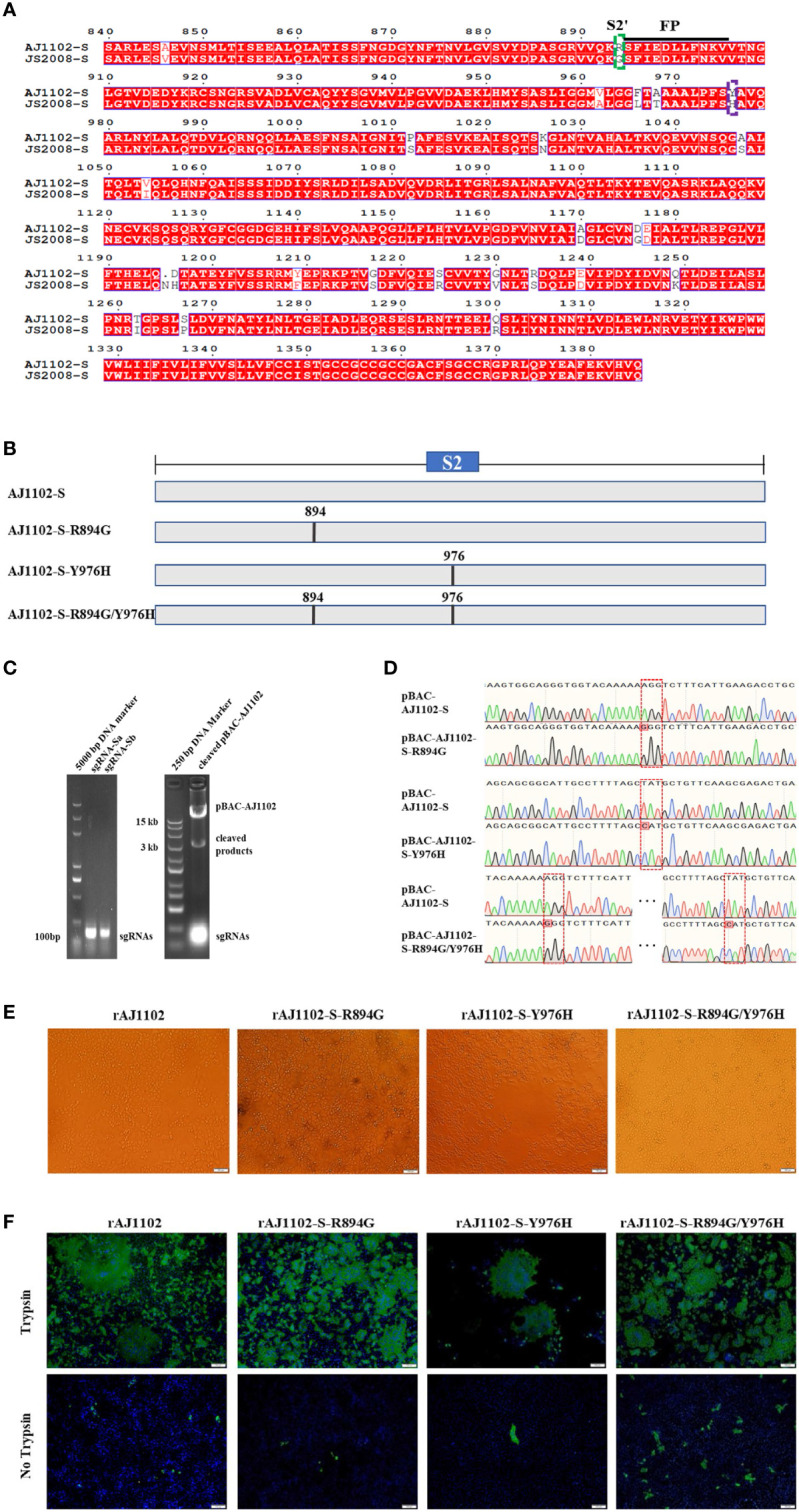
Sites R894 and Y976 in S protein are not determinants of trypsin dependence of PEDV strain AJ1102. **(A)** Amino acid alignment of S2 domains of AJ1102 and JS2008. Dotted green and purple boxes represent the S2′ site R894 and Y976, respectively. Black underline indicates the sequence of the fusion peptide (FP). **(B)** Schematic overview of the construction of recombinant BAC plasmids with a single point mutation (R894G or Y976H) or a double mutation (R894G/Y976H) in AJ1102 S protein. **(C)** Agarose gel electrophoresis of sgRNA-a and sgRNA-b and linearized pBAC-AJ1102 *in vitro*. **(D)** Identification of the recombinant BAC plasmids with amino acid mutations (pBAC-AJ1102-S-R894G, pBAC-AJ1102-S-Y976H, and pBAC-AJ1102-S-R894G/Y976H) with DNA sequencing. Red dotted boxes indicate amino acid mutation sites. **(E)** Observation of CPEs in Vero cells infected with different recombinant PEDVs at 24 hpi in the presence of trypsin. Scale bars, 100 μm. **(F)** Immunofluorescent assay of Vero cells infected with different recombinant PEDVs (MOI = 0.1) in the presence or absence of trypsin. Scale bars, 100 μm.

Previous studies have suggested that the S2′ site and Y976/977 of PEDV S might be the determinants of PEDV trypsin-independent passage (26, 28). To investigate whether amino acid mutations at these two sites of the S protein altered the trypsin dependence of strain AJ1102, we first determined the S2′ site R894 and Y976 close to the fusion peptide or HR1 of AJ1102, respectively, with a sequence alignment (**Figures 1A, B**). We then generated the linearized plasmid pBAC-AJ1102 by the *in vitro* addition of Cas9, sgRNA-a and sgRNA-b (**Figure 1C**). Three recombinant BAC plasmids (pBAC-AJ1102-S-R894G, pBAC-AJ1102-S-Y976H, and pBAC-AJ1102-S-R894G/Y976H) with a single point mutation (R894G or Y976H) or double mutation (R894G/Y976H) in S, respectively, were successfully constructed with homologous recombination between the linearized plasmid pBAC-AJ1102 and the DNA fragments containing mutations at sites encoding R894 and/or Y976 in the S gene, as confirmed with the DNA sequencing (**Figure 1D**). After Vero cells were transfected separately with each of the three recombinant BAC plasmids, the recombinant viruses were rescued and the CPEs monitored (**Figure 1E**). These recombinant PEDVs were designated rAJ1102-S-R894G, rAJ1102-S-Y976H, and rAJ1102-S-R894G/Y976H, respectively (**Figure 1E**). To our surprise, like the rescued wild-type AJ1102 (rAJ1102), all three recombinant PEDVs induced extensive syncytium formation in Vero cells in the presence of trypsin (**Figure 1F**), demonstrated with an IFA. However, rare infections of rAJ1102-S-R894G, rAJ1102-S-Y976H, and rAJ1102-S-R894G/Y976H were observed in the absence of trypsin (**Figure 1F**). These results confirmed that the mutation of R894 and/or Y976 does not alter the trypsin dependence of strain AJ1102, suggesting that R894 and Y976 in the AJ1102 S protein are not sites responsible for the trypsin dependence of PEDV strain AJ1102.

### 3.2 Trypsin promotes the infection of trypsin-dependent strain AJ1102, but not trypsin-independent strain JS2008

**Figure 2:**
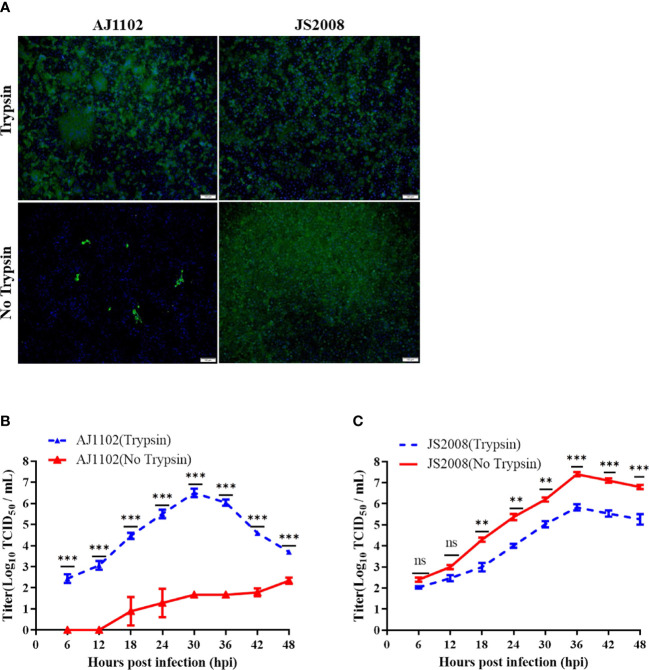
Growth characteristics of strains AJ1102 and JS2008 with and without trypsin. **(A)** Vero cells were infected with AJ1102 or JS2008 at a multiplicity of infection of 0.1 in the presence or absence of trypsin for 24 h, followed by IFA to detect viral infection. Scale bars, 100 μm. **(B, C)** Growth kinetics of AJ1102 **(B)** and JS2008 **(C)** in Vero cells in the presence or absence of trypsin. ns, not significant; **P < 0.01, ***P < 0.001.

Because our results showed that mutations at R894 and/or Y976 of the S protein did not alter the trypsin dependence of strain AJ1102, we constructed a chimeric virus by replacing the S gene of strain AJ1102 with that of a classic trypsin-independent PEDV isolate, JS2008, of genotype G1. A previous study suggested that trypsin is not essential for the propagation of JS2008 in cell culture (34). Therefore, we characterized the growth of strains AJ1102 and JS2008 in the presence and absence of trypsin with IFA and a multistep growth curve assay. As shown in **Figure 2A**, only in the presence of trypsin did AJ1102 display highly efficient infection, with the formation of large syncytia in Vero cells. However, AJ1102 infection was strictly limited in the absence of trypsin (**Figure 2A**). In contrast, JS2008 more efficiently infected Vero cells in the absence of trypsin than in its presence, and it induced no obvious syncytium formation (**Figure 2A**). Consistent with the results of the IFA, multistep growth curves showed that the titers of strain AJ1102 in the presence of trypsin were higher than those in the absence of trypsin during the viral infection process (**Figure 2B**). However, strain JS2008 reached a higher viral titer without trypsin than with trypsin during the viral infection process (**Figure 2C**). Furthermore, the highest titer of strain AJ1102 in the presence of trypsin was lower than that of strain JS2008 in the absence of trypsin (**Figures 2B, C**). These results indicate that strains AJ1102 and JS2008 have different trypsin dependence and that trypsin enhances the infection of trypsin-dependent strain AJ1102, but negatively affects the infection of trypsin-independent strain JS2008.

### 3.3 S2 (aa 894–1386) is required for the trypsin dependence of strain AJ1102

**Figure 3:**
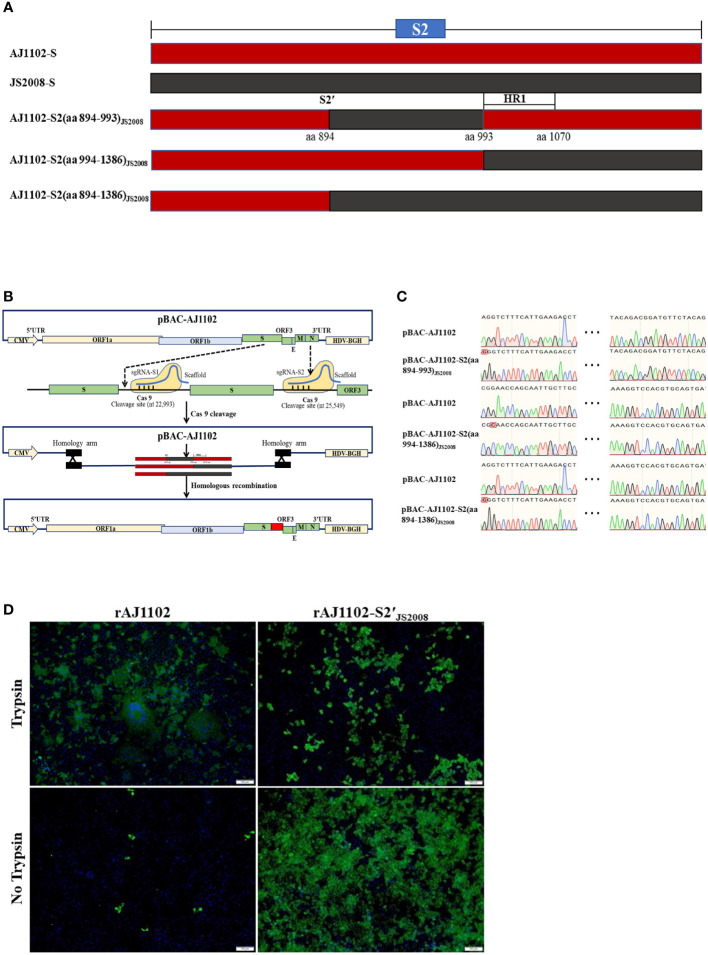
Determination of the subdomains of the S2 protein crucial for trypsin dependence of strain AJ1102. **(A)** Construction strategy of three recombinant BAC plasmids, pBAC-AJ1102-S2(aa 894-993)_JS2008_, pBAC-AJ1102-S2(aa 994-1386)_JS2008_, and pBAC-AJ1102-S2(aa 894-1386)_JS2008_, in which individual S2 subdomains of the AJ1102 S gene were replaced with the corresponding domains of JS2008. Red and black background frames represent the S2 gene sequences of strains AJ1102 and JS2008, respectively. **(B)** Schematic diagram of CRISPR/Cas9-mediated cleavage of pBAC-AJ1102 plasmid *in vitro* and homologous recombination between linearized plasmid pBAC-AJ1102 and DNA fragments of interest. **(C)** Identification of three recombinant BAC plasmids with DNA sequencing. **(D)** IFA of Vero cells infected with rAJ1102 or rAJ1102-S2(aa 894-1386)_JS2008_, designated rAJ1102-S2′_JS2008_, with or without trypsin for 24 h. Scale bars, 100 μm.

**Figure 4:**
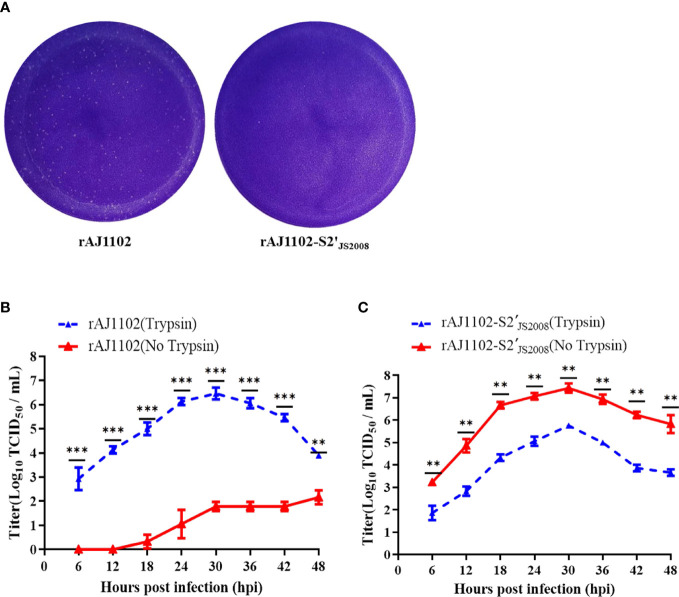
Growth characteristics of rAJ1102 and rAJ1102-S2′_JS2008_. **(A)** Plaque assays of rAJ1102 and rAJ1102-S2′_JS2008_ in Vero cells. **(B, C)** Growth kinetics of rAJ1102 **(B)** and rAJ1102-S2′_JS2008_ **(C)** in Vero cells with or without trypsin. **P < 0.01, ***P < 0.001.

Previous studies have shown that the S2 subunit is the main determinant of PEDV trypsin dependence and S2 (aa 720–892) is also not associated with the trypsin dependence (27). Therefore, we speculated that S2(aa 894-1386) is crucial domain required for the trypsin dependence of strain AJ1102 with a sequence alignment. To determine which domain in the S2 (aa 894-1386) subdomain is responsible for this trypsin dependence, we divided it into three parts: S2 (aa 894–993), S2 (aa 994-1386), and S2 (aa 894-1386) domains (**Figure 3A**). Three recombinant BAC plasmids, pBAC-AJ1102-S2(aa 894-993)_JS2008_, pBAC-AJ1102-S2(aa 994-1386)_JS2008_, and pBAC-AJ1102-S2(aa 894-1386)_JS2008_, in which one of the three S2 domains of the AJ1102 S gene was replaced with the corresponding domain of JS2008, were successfully constructed using the previously established method for the rapid manipulation of the PEDV genome with the CRISPR/Cas9 technology (36). They were confirmed with DNA sequencing (**Figures 3B, C**). However, after Vero cells were transfected with each of the three recombinant BAC plasmids, only one chimeric virus, in which the S2 (aa 894–1386) domain was replaced with the corresponding domain of JS2008, designated rAJ1102-S2′_JS2008_, was successfully recovered. Unlike rAJ1102, rAJ1102-S2′_JS2008_ achieved trypsin-independent infection without syncytium formation, causing extensive cell death (lysis) without cell fusion (**Figure 3D**). Interestingly, in the presence of trypsin, the typical plaque morphology of the rAJ1102-infected Vero cells was observed, whereas the cells infected with rAJ1102-S2′_JS2008_ did not form plaques (**Figure 4A**). We also examined the growth curves of rAJ1102 and rAJ1102-S2′_JS2008_ in the presence and absence of trypsin. As expected, the growth phenotypes of rAJ1102 and rAJ1102-S2′_JS2008_ were similar to that of the wild-types AJ1102 and JS2008, respectively (**Figures 4B, C**). Recombinant strain rAJ1102-S2′_JS2008_ reached a higher titer without trypsin than the titer of strain AJ1102 in the presence of trypsin during the viral infection process (**Figures 4B, C**). Taken together, these results demonstrate that S2 (aa 894-1386) is the key domain responsible for the trypsin dependence of strain AJ1102.

### 3.4 Immunogenicity of recombinant PEDVs in piglets

**Figure 5:**
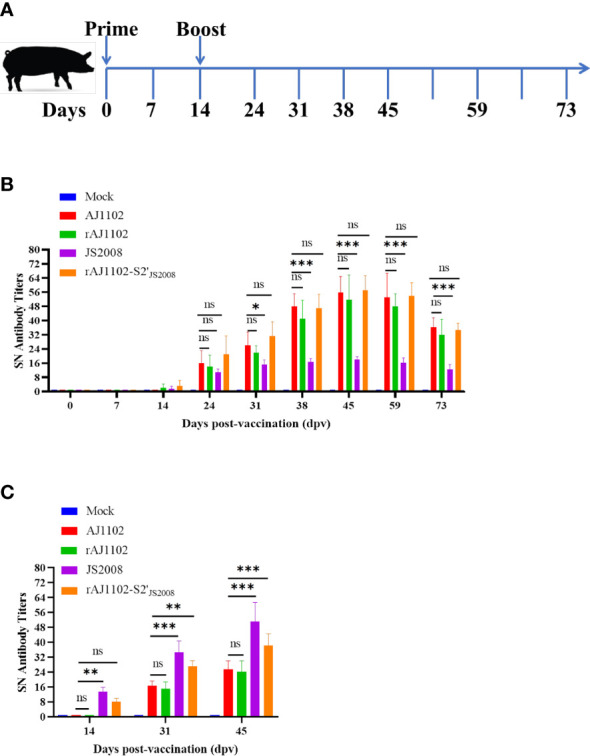
Evaluation of immunogenicity of recombinant PEDVs in piglets. **(A)** Schematic overview of animal experiments, including immune procedures and specimen collection schedule. **(B, C)** Detection of specific neutralizing antibody levels against strain AJ1102 **(B)** or JS2008 **(C)** in the sera of piglets immunized with wild-type or recombinant PEDVs on different days postvaccination. ns: not significant; *P < 0.05, **P < 0.01, ***P < 0.001.

To investigate whether the altered trypsin independence of recombinant PEDV rAJ1102-S2′_JS2008_ affects its immunogenicity and whether rAJ1102-S2′_JS2008_ provides cross-immune protection against both PEDV AJ1102 and JS2008 infections, we performed animal experiments, as described in the Material and Methods. Briefly, five groups of 28-day-old piglets, born from TGEV- and PEDV-negative sows, were vaccinated intramuscularly with 2 mL of inactivated wild-type or recombinant PEDV (10^6.5^ TCID_50_/mL) or of DMEM as a control group and then with a booster immunization 2 weeks later. Serum samples were collected at the indicated days postvaccination (dpv) and the SN titers against AJ1102 and JS2008 were determined (**Figure 5A**). Low SN titers against AJ1102 or JS2008 were detected at 14 dpv in the serum of piglets vaccinated with the different inactivated PEDVs, and increased rapidly to the average peak titer at 45 dpv (**Figures 5B, C**). Strains rAJ1102-S2′_JS2008_, AJ1102, and rAJ1102 induced similar levels of neutralizing antibodies against strain AJ1102. However, strain JS2008 induced the lowest level of neutralizing antibodies against AJ1102 at 0–73 dpv (**Figure 5B**). Based on the results shown in **Figure 5B**, we determined the neutralizing antibody titers in the sera of piglets vaccinated with the different inactivated PEDVs against JS2008 strain at 14, 31, and 45 dpv. As shown in **Figure 5C**, strain JS2008 induced the highest level of neutralizing antibodies against JS2008 on all dpv tested. The level of neutralizing antibodies induced by rAJ1102-S2′_JS2008_ against JS2008 strain was second highest. However, strains AJ1102 and rAJ1102 induced similar and the lowest levels of neutralizing antibodies against strain JS2008 on the same dpv. These results show that rAJ1102-S2′_JS2008_ induced neutralizing antibodies against PEDV strains AJ1102 and JS2008.

## 4 Discussion

The requirement of exogenous trypsin for PEDV proliferation makes it difficult to isolate and culture this virus, and to achieve high viral titers *in vitro*, increasing both the cost and complexity of vaccine production. Therefore, the construction of trypsin-independent PEDV strains may facilitate the development of novel vaccines, probably genetically engineered vaccines. In this study, we have demonstrated that the S2 (aa 894-1386) domain is the determinant of trypsin dependence of PEDV strain AJ1102. The chimeric virus rAJ1102-S2′_JS2008_ induced neutralizing antibodies against both PEDV strains AJ1102 and JS2008.

Although trypsin is necessary for the optimized propagation of most PEDV isolates, it complicates the production of PEDV vaccines. Therefore, the mechanism of trypsin activity in the context of PEDV infection has become a hot topic. Whether the S2′ site of the PEDV S protein is a potential cleavage site and trypsin-dependence determinant is currently controversial (26–28). Moreover, another amino acid site (Y976/977) of PEDV S protein has been reported to be a potential determinant of the PEDV trypsin-independent phenotype (24, 26). In this study, we have demonstrated that recombinant AJ1102 with a single point mutation at R894G or Y976H or a double mutation at these sites (R894G/Y976H) still required trypsin to effectively infect cells (**Figure 1**), indicating that the R894 and Y976 sites of the AJ1102 S protein are not critical for the trypsin dependence of PEDV. However, we cannot exclude the possibility that Y976 is a determinant of the viral trypsin-independent phenotype in other PEDV strains. We speculate that some discrepancies in the key site(s) of PEDV trypsin independence reported previously may be attributable to the analysis of different PEDV strains. Li et al. demonstrated that creating an artificial furin cleavage site at the S2′ position allowed trypsin-independent PEDV infection (38), suggesting that cleavage upstream from the fusion peptide is a necessary requirement for the fairly complex activation of the coronavirus S protein. Tan et al. reported that trypsin-enhanced infection of PEDV is determined by the S2 subunit of the S protein (27). Our results showed that the S2 (aa 894–1386) domain of the PEDV AJ1102 S protein is responsible for the trypsin-independent phenotype. This is the smallest subdomain of the S protein reported to determine PEDV trypsin dependence. Previous studies have indicated that trypsin cleavage of the S protein occurs after coronavirus–receptor binding, facilitating viral entry and release (39, 40). Many studies have shown that trypsin promotes cell–cell fusion and thus highly efficient coronavirus infection (41). The evolutionary adaptation of the coronaviruses to utilize proteases such as trypsin, cathepsin, furin, and transmembrane serine proteases to activate their S proteins may increase their cell adaptability and tropism (33, 41). However, the precise relationships between trypsin, cell–cell fusion, viral infection, and cell adaptability are still largely unclear and warrant further research.

Previous studies have shown that trypsin-independent PEDV strains often achieve higher titers than trypsin-dependent strains (23, 26), which can reduce the cost of vaccine production because the viral antigen content in these cultures is high. In this study, we generated the chimeric virus rAJ1102-S2′_JS2008_, characterized by no trypsin requirement for its propagation to high titers *in vitro*. After 30 passages, the titer of rAJ1102-S2′_JS2008_ was more than 10^8.5^ TCID_50_/mL (data not shown), indicating that rAJ1102-S2′_JS2008_ has excellent viral fitness in Vero cells. Interestingly, an immunogenicity analysis showed that rAJ1102-S2′_JS2008_ induced neutralizing antibodies against both PEDV strains AJ1102 and JS2008. Epidemiological investigations have indicated that G2 variants are the predominant PEDV strains in circulation, but the classical G1 strains are still present and circulating in China and other countries (42, 43). Therefore, rAJ1102-S2′_JS2008_ may be an important alternative novel vaccine strain because it simultaneously provides protection against both G1 and G2 PEDV infections. Importantly, it should also reduce the cost and simplify the process of PEDV vaccine production. Further research is required, including vaccination and passive protection studies of rAJ1102-S2′_JS2008_ in pregnant sows and neonatal piglets. Previous studies have shown that PEDV has four B-cell epitopes in the S protein: the core neutralizing epitope (COE) (aa 499–638), SS2 (aa 748–755), and SS6 (aa 764–771) in the S1 subunit and epitope 2C10 (aa 1368–1374) in the S2 subunit (44–47). Many researchers have developed PEDV subunit vaccines based on COE expressed in recombinant lactobacilli, *Bacillus subtilis*, tobacco plants, and *Salmonella* flagellin (48–51). rAJ1102-S2′_JS2008_ containing only the COE, SS2, and SS6 epitopes produced similar levels of neutralizing antibodies directed against strain AJ1102, suggesting that the COE, SS2, and SS6 epitopes are the most important for variant PEDV strains, whereas the 2C10 epitope is not. In contrast, rAJ1102-S2′_JS2008_ containing the 2C10 epitope also induced a high level of neutralizing antibodies against strain JS2008, although the antibody level was lower than that induced by strain JS2008. We speculate that the 2C10 epitope, as well as the COE, SS2, and SS6 epitopes, plays a crucial role in the induction of neutralizing antibodies by JS2008. However, these speculations remain to be investigated.

In conclusion, we confirmed that the S2 (aa 894–1386) domain is responsible for the trypsin dependence of PEDV strain AJ1102. We also generated the chimeric PEDV, rAJ1102-S2′_JS2008_, which not only proliferated effectively in the absence of trypsin but also induced neutralizing antibodies against both AJ1102 and JS2008, suggesting that rAJ1102-S2′_JS2008_ has potential utility as a vaccine candidate based on its obvious advantages.

## Data availability statement

The original contributions presented in the study are included in the article. Further inquiries can be directed to the corresponding authors.

## Ethics statement

All procedures involving animal experiments were reviewed, approved, and conducted in strict accordance with the Animal Experimental Ethical Inspection of Laboratory Animal Centre, Huazhong Agricultural University (Ethics Approval Number: HZAUSW-2023-0001).

## Author contributions

ML, SX, and LF conceived the research project, designed the study, directed the entire research activities. ML and PF interpreted data and wrote the manuscript. ML, YZ, and YF performed experiments. All authors contributed to the article and approved the submitted version.
